# Neuroethics: what the study of brain disorders can tell about moral behavior

**DOI:** 10.3934/Neuroscience.2021029

**Published:** 2021-10-25

**Authors:** Carmelo M Vicario, Chiara Lucifora

**Affiliations:** Dipartimento di Scienze Cognitive, Psicologiche, Pedagogiche e degli studi culturali Università di Messina, via concezione, Messina, Italy

**Keywords:** Neuroethics, clinical models, morality, disgust

## Abstract

The growing interest in the study of morality has led to the birth of a new discipline in the field of moral philosophy called Neuroethics, a multidisciplinary approach that aims to combine philosophy and neuroscience. In this editorial, we explored the relevance of clinical models affected by neurological/psychiatric disorders to learn more about mechanisms sub-serving ethical behaviour at neural and cognitive level.

The growing interest of philosophers to neuroscience has promoted the birth of a new discipline in the field of ethics called *Neuroethics*.

The innovative side of this discipline is the adoption of neuroscientific methods to explain how, and which neural processes subserve ethical reasoning. However, it should be clarified that the mission of Neuroethics is not limited to the investigation of neurobiological correlates of moral behavior, but it also includes questions about the moral implications of neuroscientific methods. In this regard, it is useful the terminological distinction by Adina Roskies between “*Neurosciences of Ethics*” and “*Ethics of Neurosciences*” [1].

Regarding the “*Neuroscience of Ethics*”, some interesting insight can be derived from the study of clinical populations affected by neurological and/or psychiatric disorders. A paradigmatic case, which is probably the first one reported in the literature, was described by Harrlow [2] with the patient Phineas Gage. As result of a work accident, which made this patient victim of a severe brain injury involving the medial and orbital regions of the frontal lobe, Phineas Gage had become an impulsive, violent and profane man. Damasio [3] suggested that a lesion of the ventromedial prefrontal cortex (vmPFC) leads to the loss of the influence of ethics principles and the emotional appraisal ​​typically involved in the evaluation of moral outcome such as the discrimination of good from bad. Moreover, patients affected by a damage of the vmPFC, were unable to correct or control harmful behaviors and/or unusual reactions while facing moral outcomes, such as those provided through the “Iowa Gambling” Task [4], a psychological task developed to measure “real world” decision-making deficits in patients with damage to the prefrontal cortex [5]. According to Damasio [3], it is not the reasoning ability of these patients that is affected but their emotions, intended as somatic markers used by the brain to quickly and unconsciously filter options with important positive or negative emotional consequences.

If the case of Phineas Gage highlights the importance of the frontal lobe in moral behavior, subsequent studies involving healthy participants through the use of neurophysiological techniques, such as functional magnetic resonance imaging and non-invasive brain stimulation methods, have revealed a wider and much more complex neural network. Among these regions it should be mentioned the cingulate cortex, a neural structure considered important in mediating the conflict between the emotional and rational component of moral reasoning [6]; the insula, a neural structure of central importance in the elaboration of interoceptive states (e.g. [7],[8]), which seems to be involved in the elaboration of the affective component of the sense of iniquity [6],[9]; and the basal ganglia such as the subthalamic nucleus, which is involved in the evaluation of morally related conflicting situations [10].

Turning back to the relevance of clinical models in the field of Neuroethics, it should be mentioned the case of movement disorders such as Parkinson's syndrome, Huntington's chorea, and Tourette's syndrome, which are characterized by a reduced sensitivity towards ethical violations (for a review see [11],[12],[13]. Moreover, an important contribute comes from the examination of psychiatric syndromes such as obsessive-compulsive disorder (OCD) [11],[14] and Depression [15], which are characterized by a high sensitivity to ethical violations. It is interesting to note that all syndromes mentioned above are affected by similar anatomo-functional alteration of neural structures (Insula, Cingulate Cortex, Basal Ganglia) involved in moral processing (see [11] for a review). Furthermore, for all these syndromes it is documented an altered representation/perception of disgust (from core disgust to social disgust).

Disgust can be explained as a negative emotion towards specific stimuli that leads to avoid them [16]. Since several years there is a debate about a possible relationship between disgust and morality. Pizarro and colleagues [17] developed three different principles to explain this relationship. In the first case, it is possible to speak about the experience of disgust as a consequence of moral disapproval (e.g., [18]). In the second case, disgust becomes an instrument to prime (amplify) moral rejection. For example, it was shown that inducing disgust leads people to have harsher moral judgments (e.g.,[19]). Moreover, the lower the disgust sensitivity the lower the disapproval of ethical violations (e.g., [20]). In the third case, disgust is understood as an engine to influence the judgment of morally neutral acts. According to the second principle, in Parkinson's Syndrome [21],[22] or Huntington's chorea [23],[24], the sensitivity to core disgust is reduced compared to controls. On the other hand, a higher sensitivity to disgust has been reported in patients affected by OCD [25] and depression (e.g.,[26]).

In Tourette's syndrome, we recently documented higher disgust sensitivity compared to controls [13]. This contrasts the greater tolerance of unethical behaviors in TS adolescents compared to controls, as the general literature which documents higher moral disapproval in individuals with higher disgust sensitivity [20],[27]. However, the reduced moral disapproval of ethical violations in TS is consistent with prior evidence of increased impulsivity in this clinical population [28], which is associated with reduced moral disapproval in healthy humans [29].

The evidence provided by the study of ethical behavior in clinical models supports the neo-sentimentalist perspective, which postulates a close relationship between disgust and morality (e.g.,[30]). However, it should be clarified that a distinction exists between “emotional appraisal” and “utilitarian appraisal” in the judgment of moral outcomes [31]. From a critical analysis [9] of the fMRI study by Hutcherson et al. [31], it was outlined that the neural circuits classically involved in the processing of disgust only overlap those involved in the “emotional appraisal” of moral outcomes. On the other hand, the neural circuit reported in response to “utilitarian appraisal” of moral topics is clearly independent from the one associated with disgust processing. This suggests that the influence of disgust on morality may be mainly involved in (if not limited to) the emotional evaluation of moral outcomes, instead of being considered the general root of morality.

In summary, these results provide a contribute to the current debate between the neosentimentalist and rationalist perspective of morality by suggesting that the influence of disgust on ethics behavior is related with the affective appraisal of this human experience.

In conclusion, the study of ethics through clinical models affected by neural disorders and, more generally, through the adoption of neuroscientific methods represents a valuable approach to learn more about the origin of the noblest faculty of the human being.
